# Safe doubling of ventilator capacity: a last resort proposal for last resorts

**DOI:** 10.1186/s13054-020-02945-z

**Published:** 2020-05-14

**Authors:** J. Geoffrey Chase, Yeong Shiong Chiew, Bernard Lambermont, Philippe Morimont, Geoffrey M. Shaw, Thomas Desaive

**Affiliations:** 1 Department of Mechanical Engineering, University of Canterbury, Christchurch, New Zealand; 2 School of Engineering, Monash University, Bandar Sunway, Malaysia; 3 Department of Intensive Care, University of Liège hospital, Liège, Belgium; 4 Department of Intensive Care, Christchurch Hospital, Christchurch, New Zealand; 5 GIGA-In silico medicine, University of Liège, Liège, Belgium

*The best way to ventilate two patients on a single ventilator is simply not to do it: The Authors*

In light of the COVID-19 pandemic, this common-sense approach was recently clarified in a SCCM-ASA-AARC-AACN-ASPF-CHEST consensus statement on the Society of Critical Care Medicine (SCCM) website [1]: ‘We recommend that clinicians do not attempt to ventilate more than one patient with a single ventilator while any clinically proven, safe, and reliable therapy remains available (ie, in a dire, temporary emergency)’ [1].

The current situation in several European countries and states in the USA is a ‘dire emergency’. Physicians have been, or may be, asked to make difficult choices in the face of ventilator shortages [2]. Nevertheless, if you are faced with a decision to ventilate two patients at once, or deny care to one, we believe we can propose the next best way.

The SCCM recommendation [1] addresses a series of popular Internet concepts with multiple patients breathing in-parallel [3, 4]. In-parallel is a critical point, as inspiration and expiration all take place at the same time, so there is thus no change to respiratory rate (RR) and tidal volume or driving pressure are adjusted for the number of patients. All of these add risk over over-/under-ventilating patients and causing harm [1].

Instead, we recommend a multiplex in-series breathing approach to double (2-for-1) the patients on a ventilator (Fig. 1). In-series breathing means only 1 circuit volume (split between patients) is active at a time, but each patient’s inspiratory effort is singular. This approach addresses the limitations of shared, in-parallel breathing in the SCCM statement, where Table 1 addresses in detail each consensus statement concern [1].

**Fig. 1:**
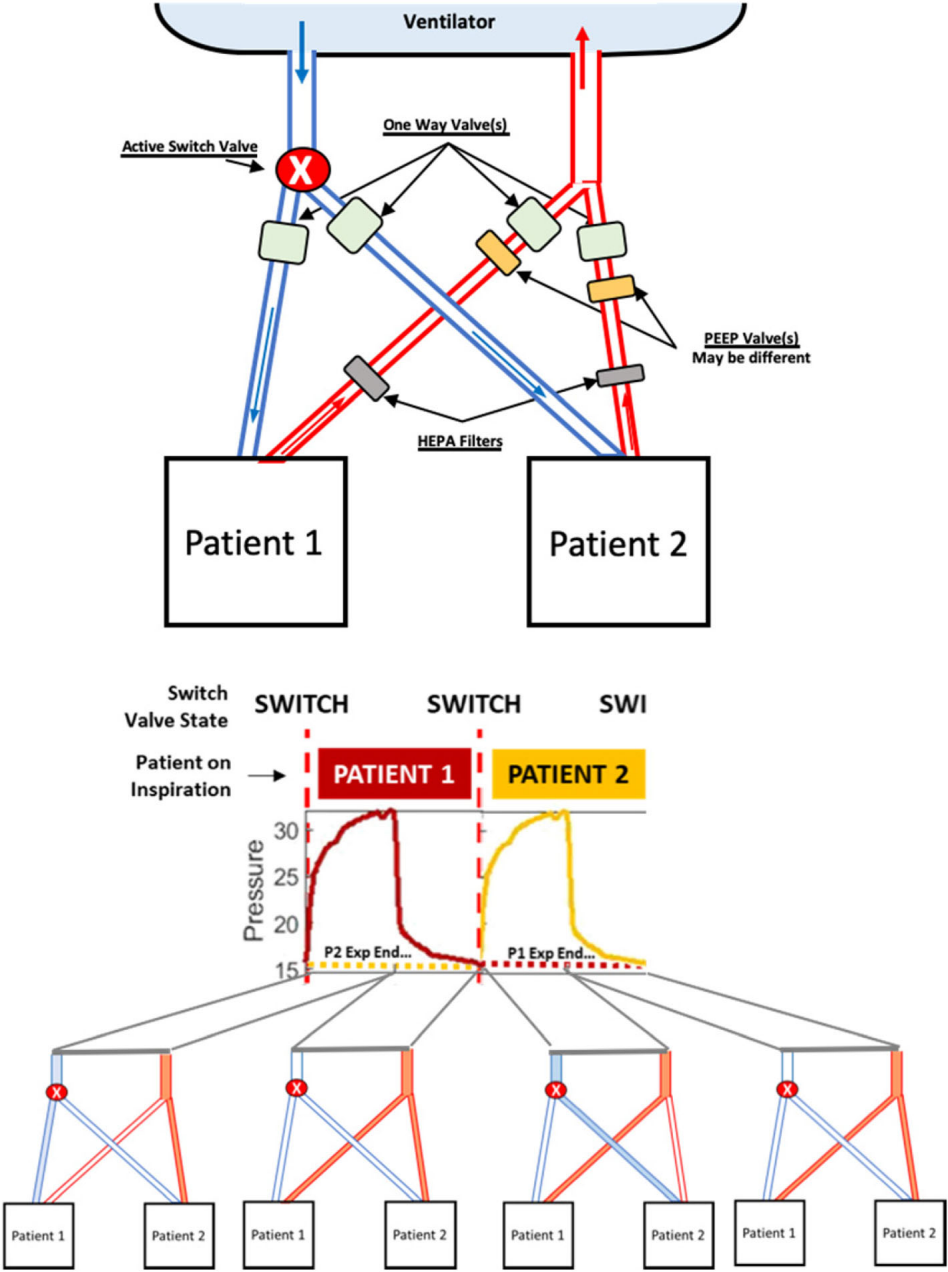
Top: Schematic of a proposed in-series breathing circuit for two patients using an active inspiration valve. Bottom: Resulting ventilation waveforms and active (filled in) and in-active (not filed) inspiratory and expiratory circuit lines at any given 2-s period for 2 × 4-s breaths, one by each patient. The ventilator will display the given patient data in each breath. Patients are colour-coded for clarity and show how end-expiration of one patient overlaps inspiration and initial expiration of a second patient, although using different parts of the circuit

**Table 1:** Specific reasons and multiplex series approach mitigation: The SCCM/ASA/AARC/AACN/ASPF/CHEST consensus statement [1] strongly recommends against parallel breathing and ventilation of patients; this poses a wide range of valid criticisms. We address these criticisms in terms of the serial ventilation approach/concept presented *VC* volume-controlled, *PC* pressure-controlled ventilation, *PEEP* positive end-expiratory pressure, *PIP* peak inspiratory pressure, *Vt* tidal volume

Consensus statement specific critiques (SCCM/ASA/AARC/AACN/ASPF/CHEST):	Mitigation by serial ventilation approach vs parallel
Volumes would go to the most compliant lung segments.	Serial breathing ventilates a single lung at a time, and thus, using a volume-controlled (VC) mode, lung compliances are not ‘mixed’ and do not create this same, critical problem. A pressure controlled (PC) mode will also be separated. Critically, in all modes, each lung responds individually to the inputs.
Positive end-expiratory pressure, which is of critical importance in these patients, would be impossible to manage.	PEEP can be individually set using PEEP valves on the expiratory circuit and putting PEEP = 0 on the ventilator. These valves are commonly available and some come with multiple settings. Thus, PEEP may also be individualised
Monitoring patients and measuring pulmonary mechanics would be challenging, if not impossible.	Patients are split in serial breathing so inspiration does not overlap, and any monitoring present would monitor each inspiratory portion (at least) separately. Monitoring mechanics would depend on the ventilator interface and monitoring algorithms used, thus the displayed patient-specific parameters would be averaged. However, clinicians could still examine breath by breath waveforms or PV loops. PIP or Vt alarm limits could still be used as these are based on safety settings determined for a population of patients, rather than individual patients. Again, these outcomes are enabled by separating inspiration for both patients.
Alarm monitoring and management would not be feasible.	See above, again by separating patient inspiration segments in serial ventilation this issue is mitigated.
Individualised management for clinical improvement or deterioration would be impossible.	PC driving pressure and VC tidal volume would have to be the same as ventilators currently do not have the capability to enable alternating breath settings. Clinical judgement would determine which one of the ventilation is most appropriate for this situation. Where there are significant differences in compliance, a volume-controlled mode may be preferable. However, PEEP would be individualised via separate PEEP valves. These PEEP valves could also be made active if desired, or set manually similarly to changing PEEP on a ventilator, but for each patient.
In the case of a cardiac arrest, ventilation to all patients would need to be stopped to allow the change to bag ventilation without aerosolizing the virus and exposing healthcare workers. This circumstance also would alter breath delivery dynamics to the other patients.	In this case, the patient still on the ventilator can be restored to a 1 patient, 1 ventilator standard use, after the other patient is disconnected. Alternatively, a rubber bag (test lung) could be swapped in while the arrested patient is being hand ventilated during CPR. This would not involve having to make changes to the ventilator settings, which would create cognitive overload in the event of a cardiac arrest.
The added circuit volume defeats the operational self-test (the test fails). The clinician would be required to operate the ventilator without a successful test, adding to errors in the measurement.	Self-testing can be carried out in the usual manner. There is no added circuit volume as individual breaths are within usual physiological limits and therefore not vulnerable to errors of extrapolation created by connecting patients in parallel.
Additional external monitoring would be required. The ventilator monitors the average pressures and volumes.	In serial breathing, each breath would be presented. The clinician would have to know to identify each patient by examining their breathing directly, to know which waveform or PV loop corresponds to a particular patient.
Even if all patients connected to a single ventilator have the same clinical features at initiation, they could deteriorate and recover at different rates, and distribution of gas to each patient would be unequal and unmonitored. The sickest patient would get the smallest tidal volume and the improving patient would get the largest tidal volume.	Since each patient is separated, there is less need for matching compliance or resistance, the latter of which would be similar. Specifically, for the following: PC: driving pressure would have to be the same. However, a resistor with known pressure drop can be added to one of the two inspiratory circuits to reduce driving pressure for one patient. Tidal volume alarming would still be feasible and ventilator controlled to avoid injury or damage. VC: tidal volume would be the same for both patients, where we would recommend setting tidal volume for the smaller of two patients in ml/kg; however, a vast difference could be problematic requiring some light matching by approximate size. PIP alarms and limits would still be applicable and ventilator controlled.
The greatest risks occur with sudden deterioration of a single patient (e.g. pneumothorax, kinked endotracheal tube), with the balance of ventilation distributed to the other patients.	Patients are ventilated separately so changes in patient condition, resulting in tidal volume changes (during PC) or peak pressure changes (during VC) would be notable on the monitor and ventilator set limits and alarms would still work be useful.
Finally, there are ethical issues. If the ventilator can be lifesaving for a single individual, using it on more than one patient at a time risks life-threatening treatment failure for all of them.	The best way to ventilate 2 patients on 1 ventilator *is not to do it*! Given the exigency of no other alternative, we propose this method is currently the *next best way*.

Instead of the same RR and higher tidal volume or driving pressure, in-series breathing doubles the RR and keeps the other ventilator settings the same. One patient breathes in, while the other breathes out. With typical I:E ratios around 1:3, there is also shared expiration time when neither is breathing in (Fig. 1). As with other proposals [3], driving pressure can be modified by added resistors in the inspiratory circuit, and PEEP can be customised with in-line expiratory PEEP valves (Table 1).

This in-series approach ensures breath-by-breath ventilation parameters of each patient are displayed to ensure monitoring and safety are maintained. Staff can thus be assured more (or less) compliant patients are not over- (under-) ventilated, both of which could result in harm if ventilated in-parallel, where monitoring individual patients is not possible. Finally, one-way expiratory valves and filters prevent rebreathing and cross-contamination.

Clinically, we would suggest pressure-control modes, where driving pressures are easily customised per-patient with resistors, and are more commonly used, currently. This choice allows customised PEEP and driving pressure for each patient effectively as if they were ventilated separately.

This setup requires an active valve to switch between patients (Fig. 1), comprising a pressure sensor at the single end of a y-splitter on the inspiration circuit, and two active valves at the outlet. It uses measured inspiratory pressure to switch the inspiratory circuit from one patient to the other after inspiration (as pressure drops). The active y-splitter valve thus allows flow down only one inspiratory path per ventilator-supplied breath (at 2xRR). The components, sensors, and computation are low-cost and easily 3D printable by hospital bioengineers or others.

However, nothing is perfect. This approach is not suited for spontaneous, triggered breathing, which cannot be synchronised nor limited. In addition, it cannot be used if I:E < 1, or if a patient’s respiratory mechanics are such they will receive inadequate minute ventilation within the time for each allocated breath.

## Data Availability

Not applicable

## References

[1] SCCM, et al., Consensus Statement on Multiple Patients Per Ventilator. SCCM Website (https://www.sccm.org/Disaster/Joint-Statement-on-Multiple-Patients-Per-Ventilato), 2020. Accessed 13 Apr 2020.

[2] Truog RD, Mitchell C, Daley GQ. The Toughest Triage - Allocating Ventilators in a Pandemic [published online ahead of print, 2020 Mar 23]. N Engl J Med. 2020;10.1056/NEJMp2005689. 10.1056/NEJMp2005689. Accessed 13 Apr 2020. 10.1056/NEJMp2005689 32202721

[3] Pinson, H., A better way of connecting multiple patients to a ventilator, Medium.com, Editor. 2020, Medium.com: Medium.com (https://medium.com/@pinsonhannah/a-better-way-of-connecting-multiple-patients-to-a-single-ventilator-fa9cf42679c6). P. Blog Post by Hannah Pinson.

[4] Differential Multiventilation International Working Group. Differential Multiventilation. Differential Multiventilation [https://www.differentialmultivent.org/] 2020 [cited 2020 April 13, 2020]; Available from: Differential Multiventilation.

